# Symptoms of internet gaming disorder among male college students in Nanchong, China

**DOI:** 10.1186/s12888-022-03778-6

**Published:** 2022-02-22

**Authors:** Fang Liu, Hongjie Deng, Qin Zhang, Quan Fang, Boxi Liu, Dan Yang, Xiaobin Tian, Xin Wang

**Affiliations:** 1 School of Public Health, China Medical University, No.77 Puhe Road, Shenyang North New District, Shenyang, 110122 Liaoning China; 2 Teaching Affairs Department, North Sichuan Medical College, No.234 Fujiang Road, Nanchong, 637000 Sichuan China; 3 School of Health Management, China Medical University, No.77 Puhe Road, Shenyang North New District, Shenyang, 110122 Liaoning China; 4 Nanchong Physical and Mental Hospital (Nanchong Sixth People’s Hospital), No.99 Jincheng Street, Yingshan County, Nanchong, 637000 Sichuan China; 5 Department of Preventive Medicine, North Sichuan Medical College, No.234 Fujiang Road, Nanchong, 637000 Sichuan China

**Keywords:** Internet gaming disorder, Symptoms, Depressive symptoms, Family and peer support, Chinese college students

## Abstract

**Background:**

This study aimed to evaluate the presence of symptoms of Internet Gaming Disorder (IGD) and examined associations between IGD and depressive symptoms, family and peer support among male college students in Nanchong, China.

**Methods:**

A cross-sectional study was conducted among 2533 male students in three colleges. Background characteristics, depressive symptoms, family and peer support and IGD information were collected. Binary logistic regression was performed to access the relationship between variables and IGD. PROCESS macro was used to examine the mediation analysis of family and peer support on the relationship between depressive symptoms and IGD.

**Results:**

The estimated presence of symptoms of IGD was 11.6%. The most commonly endorsed items were escapism, continuation and preoccupation both among total participates and the IGD group. In the binary logistic regression, general expenditure per month, depressive symptoms, and family and peer support revealed their significance in associations with IGD. Adjusted for the significant background variable, depressive symptoms and family and peer support remained significance. Additionally, family and peer support would attenuate the relationship between depressive symptoms and IGD.

**Conclusions:**

This study found that one in ten male college students reported clinically significant IGD symptoms, which indicate that IGD is an important public health problem in Nanchong, China.

## Introduction

Internet Gaming Disorder (IGD), defined as an excessive activity involving the persistent and recurrent internet use to play videogames, resulting to considerable impairment or distress over a period of 1 year [1]. Individuals with IGD experience symptoms similar to those who developed substance dependence; these symptoms include preoccupation with gaming, developing a tolerance, withdrawal symptoms, unsuccessful attempts to stop, escape from bad moods, and jeopardizing significant relationships or opportunities because of gaming [2]. IGD has emerged as a pervasive and serious public health threat worldwide [2].

Data from the Cyberspace Administration of China, the number of Internet users in China has reached 989 million as of December 2020, among which, students accounted the most (21%) [3]. With this soaring number of Internet users, the problem of IGD has attracted high attention from society. Evaluating the presence of symptoms of IGD provide essential information for schools, health policy-makers and society to take effective measures to promote students’ mental health. Though studies on the presence of symptoms of IGD have been increasing, some research gaps remain. Most of the studies aimed at middle or high school students [4–6], adolescents [7, 8] or general population [2], only limited investigations focused on college students. In fact, more attention should be paid to the problem of IGD among college students. Compared with other segments of society, college students are more vulnerable to Internet addiction as they have easier access to the Internet, and prefer to pursue relationships through online activities [9]. Moreover, few works examined the less developed regions. As is well known, China obtains a vast territory, and the development gap between eastern, central and western regions is huge, each region with their unique particularities and limitations, which needs to adjust measurements based on local conditions. Compare to developed areas, less developed western areas demonstrate not only technological gaps, but also educational resource gaps, which needs to pay more attention.

In addition to the presence of symptoms of IGD, a careful assessment of risk and protective factors of IGD is also important, which is essential for prevention and intervention programs. Depressive symptoms are usually regarded as negative emotional experiences such as those which might ensue after a series of negative life events [10]. It is considered as one of the most common psychological conditions [11]. Evidence revealed that Internet addiction and depression among adolescents is likely to form a vicious circle, that depressed adolescents would have excessive Internet use as a means of alleviating their negative mood, while negative consequences of such excessive use (e.g., social isolation) would further intensify depressive symptoms [12]. Social support is a basic psychological need that contributes to personal health [13]. As for Chinese college students, most of them are living independently from their parents for the first time, and their main social environment is composed of peers [13]. Support from parents and peers appear to be a very important aspect of social support that the students had embraced, and it plays significant roles in students’ socialization, behavioral development, and mental health [8]. It has been previously observed that higher social support is associated with fewer depressive symptoms [14, 15], and well received social support can limit the prevalence of adolescents’ online addiction [16]. As a result, social support may attenuate the relationship between depressive symptoms and IGD in college students. Nevertheless, it has not been explored yet.

Taken together, the purpose of our study was to evaluate the presence of symptoms of IGD among male college students in Nanchong City, to explore risk and protective factors of IGD. This study also aimed at testing whether family and peer support would attenuate the relationship between depressive symptoms and IGD.

## Methods

### Study settings

This study was conducted in Nanchong City, which is an economically underdeveloped area of western China, located in the northeast of Sichuan Province. Nanchong has jurisdiction over nine counties, while four national-level poverty-stricken counties and three provincial-level poverty-stricken counties had been removed from the poverty-stricken county list until 2019. The per capita disposable income of Nanchong accounted for only 78.77% of the national average in 2020. Shunqing District, as the center of Nanchong, was selected as the sampling areas. Shunqing District has four tertiary institutions, which include Southwest Petroleum University, China West Normal University, North Sichuan Medical College, and Nanchong Vocational and Technical College.

### Participants

A stratified cluster sampling investigation was applied to recruit participants. Three colleges with different quality rankings were randomly selected. Then, two classes were sampled from each grade level at each college. In total, twenty-four classes were selected to complete the survey. The inclusion criteria for the participants were as follows: (1) had student status in the school, (2) male, and (3) voluntarily agreed to participate. The exclusion criteria for the participants were as follows: (1) had dropped out of school, (2) female, and (3) did not agree to participate in the survey.

The sample size was calculated based on the following formula: $n=\frac{Z^2\frac{a}{2}\times p\left(1-p\right)}{d^2}$ [17].

$${Z}_{\frac{a}{2}}=1.96,\mathrm{p}=17\%,\mathrm{d}=0.02$$

We wanted to estimate the required sample size for the presence of symptoms of IGD in college students in which the marginal error of its estimate does not exceed than 2% (d = 0.02) with 95% confidence level (${Z}_{\frac{a}{2}}=1.96$). A systematic review showed that the prevalence rates of problematic online gaming ranged from 3.5 to 17% in China [18], therefore, we set the *p* = 17%. With that being said, the sample size was calculated as $n=\frac{1.96^2\times 0.17\times 0.83}{0.02^2}=1,355$. Considering of lost-to-follow-up, rejection and invalid data, we planned to increase double. Finally, the sample size was set as follows: *N* = 1355 + 1355 = 2710. At the end of the study, a total of 2625 students completed the survey. We excluded 92 invalid samples from the analysis, resulting in 2533 valid responses.

### Procedures

The survey was conducted from September 2018 to February 2019. Before data collection, standard training was provided to the survey administrators to ensure the quality of the survey. Consent and permission to administer the survey was obtained by school principals prior to data collection. During the survey, the students were instructed to sit apart and to refrain from discussing any of the question with other students. All participants were informed of the background and aims of our study. Participants provided their written informed consent to the survey, and they had the right to withdraw from the study at any time. Then, an anonymous structured questionnaire was administered to the students in the absence of teachers in classroom. The survey was approved by the ethics committee of North Sichuan Medical College.

### Measures

#### Background characteristics

The individual characteristics comprised age, grade, native residential area and general expenditure per month.

#### Depressive symptoms

Depressive symptoms were assessed using the short form of the Epidemiologic Studies Depression Scale (CESD-10). As a kind of the most commonly used self-report instruments in screening depressive symptomatology, brief CESD demonstrates adequate reliability and validity [19]. One finding confirmed that the Chinese-version CESD-10 was most appropriate for the nonclinical, general population [20]. All items were responded on a four-point Likert scale ranging from 0 = rarely or none of the time (less than 1 day) to 3 = almost or all of the time (5–7 days) [21]. The total score ranged from 0 to 30, with a higher score reflecting more depressive symptoms. As suggested, a score of 10 or above was classified as clinically relevant depressive symptoms [22]. In order to further understand the hierarchical relationship between depressive symptoms and IGD, we further divided total score into three levels: mild risk (score ranging 0–9), moderate risk (score ranging 10–19) and high risk (score ranging 20–30). The scale showed good internal reliability in the present study (Cronbach’s α = 0.65).

#### Family and peer support

Family and peer support was measured by six items consisting of three dimensions (emotional support, instrumental support, and affirmation from parents and peers), such as “Q1. How much support had you received from your parents when you needed to talk with someone or needed emotional support?”; “Q2. How much support had your received from your parents when you needed instrumental support?”; “Q3. How much support had you received from your parents when you needed appreciation?”. To access peers’ support, the wording “parents” was replaced by “peers”. Each item score ranged from 0 = none to 10 = very much. The total score ranged from 0 to 60, with a higher score reflecting much support. We divided total score into three levels: mild level (score ranging 0–20), moderate level (score ranging 21–40) and high level (score ranging 41–60). Similar measurements had been used in previous studies [5, 8, 23]. Good reliability was obtained in the present study (Cronbach’s α = 0.82). Additionally, the goodness-of-fit for the scale was shown as χ^2^/df = 1.41, GFI = 0.99, AGFI = 0.99, NFI = 0.99, TLI = 0.99, RMSEA = 0.01 in the structural equation modeling, indicating an excellent model fit.

#### Internet gaming disorder

The presence of symptoms of IGD was assessed using the diagnostic criteria in the Diagnostic and Statistical Manual of Mental Disorders Fifth Edition (DSM-5) [24]. Previous findings confirmed that the IGD symptoms proposed by the DSM-5 can be measured as a single underlying factor both among online gamers and in the general population [25]. The measure comprised nine diagnostic criteria to assess IGD tendency and identify IGD [2], and showed satisfactory psychometric properties, providing good support for its reliability and validity in China [26]. Participants were asked to indicate whether each of these symptoms (e.g., preoccupation, withdrawal, tolerance, loss of control, loss of interest in other activities, continuation, deception, escapism, and jeopardizing) described their own condition in the past 12 months (0 = no, 1 = yes). The proposed cut-point of 5 criteria was conservatively chosen in the DSM-5 [27]. A higher level of IGD symptoms indicated a higher tendency to have IGD. In the present study, the scale showed good internal reliability (Cronbach’s α = 0.76).

### Data analysis

First, descriptive statistical analyses were employed to describe the participates’ demographic profiles.

Continuous variables (age) were represented by means ± *standard* deviations. Categorical variables (grade, native residential area, general expenditure per month, depressive symptoms, and family and peer support) were described using frequencies (percentages). Subsequently, we estimated the presence of symptoms of IGD and frequency of endorsement on each DSM-5 criterion. Next, we categorized the overall sample into two subsamples (i.e., having IGD OR non) according to the cut-off point of 5 [27]. Then, we applied binary logistic regression analyses to explore the association between IGD and background characteristics, depressive symptoms, family and peer support. Results were presented as odds ratios (ORs) and 95% CIs. After controlling background characteristics, we included depressive symptoms, and family and peer support into a logistic regression model to obtain adjusted ORs (AORs) and the corresponding 95% CIs. At last, we examined the mediation analysis of family and peer support on the relationship between depressive symptoms and IGD using PROCESS macro [28] in SPSS26 with a bootstrap threshold of 5000 and model 4. Depressive symptoms were used as independent variable, family and peer support were used as mediating variable, and IGD was used as dependent variable. All analyses were performed by using SPSS26 (IBM Corp., Armonk, NY, USA) and *p* < 0.05 was considered statistically significant.

## Results

### Participants’ characteristics

The sample consisted of 2533 male college students with a mean age of (20.08 ± 1.56) years (range 16–30). As shown in Table 1, majority of the respondents were sophomore (32.6%), followed by junior (28.9%). About 39.9% came from countryside. Over half reported that their general expenditure per month were 1001–2000 of CNY (57.9%). A total of 3.2% of the participants reported they had high risk depressive symptoms and 60% reported they had high level of family and peer support.

**Table 1:** Characteristics of the participants

Variables	Total participates (***N*** = 2533)	Have IGD or not	
		No (***n*** = 2239)	Yes (***n*** = 294)
**Age, years, mean ± SD**	20.08 ± 1.56	20.08 ± 1.56	20.04 ± 1.57
**Grade, n (%)**			
Freshman	531 (21.0)	479 (90.2)	52 (9.8)
Sophomore	826 (32.6)	722 (87.4)	104 (12.3)
Junior	732 (28.9)	643 (87.8)	89 (12.2)
Senior	444 (17.5)	395 (89.0)	49 (11.0)
**Native residential area, n (%)**			
Big city	184 (7.3)	165 (89.7)	19 (10.3)
Small and medium-sized cities	697 (27.5)	635 (91.1)	62 (8.9)
Town	641 (25.3)	559 (87.2)	82 (12.8)
Countryside	1011 (39.9)	880 (87.0)	131 (13.0)
**General expenditure per month (CNY), n (%)**			
≤ 500	78 (3.1)	60 (76.9)	18 (23.1)
501–1000	851 (33.6)	753 (88.5)	98 (11.5)
1001-2000	1466 (57.9)	1314 (89.6)	152 (10.4)
≥ 2001	138 (5.4)	112 (81.2)	26 (18.8)
**Depressive symptoms (score ranging 0–30), n (%)**			
0–9	1164 (46.0)	1091 (93.7)	73 (6.3)
10–19	1288 (50.8)	1090 (84.6)	198 (15.4)
20–30	81 (3.2)	58 (71.6)	23 (28.4)
**Family and peer support (score ranging 0–60), n (%)**			
0–20	85 (3.4)	65 (76.5)	20 (23.5)
21–40	929 (36.7)	798 (85.9)	131 (14.1)
41–60	1519 (60.0)	1376 (90.6)	143 (9.4)

*SD* standard deviation, *CNY* China Yuan; 1 CNY ≈ 0.1569 USD (January 6, 2022 exchange rate)

### Presence of symptoms of IGD and criteria endorsement

Based on the cut-off point of 5, 294 out of 2533 participates were identified as having the presence of symptoms of IGD, which is estimated to be 11.6% (95% CI: 10.4,12.9%). The frequency of endorsement on each DSM-5 criterion was listed in Table 2. Among all participates, the most commonly endorsed items were escapism (793, 35.4%), continuation (460, 20.5%) and preoccupation (419, 18.7%), respectively. Among participates who had IGD, preoccupation (251, 85.4%), escapism (246, 83.7%) and continuation (238, 81.0%) were the most commonly endorsed items.

**Table 2:** Endorsement rate of each item of the DSM-5 criteria

Item description	Total participates (***N*** = 2533)		IGD (***n*** = 294)
	Yes	No	Yes
1. Feel preoccupied with Internet games.	419 (18.7%)	1820 (81.3%)	251 (85.4%)
2. Feel irritable, anxious, or sad when Internet gaming is taken away.	129 (5.8%)	2110 (94.2%)	167 (56.8%)
3. Spend increasing amounts of time on gaming to achieve satisfaction.	131 (5.9%)	2108 (94.1%)	185 (62.9%)
4. Have made unsuccessful attempts to control your participation in Internet games.	319 (14.2%)	1920 (85.8%)	219 (74.5%)
5. Have lost interests in previous hobbies and entertainment as a result of, and with the exceptions of, Internet games.	112 (5.0%)	2127 (95.0%)	164 (55.8%)
6. Continue to play Internet games excessively despite knowledge of psychosocial problems.	460 (20.5%)	1779 (79.5%)	238 (81.0%)
7. Have deceived others regarding the amount of Internet gaming.	168 (7.5%)	2071 (92.5%)	165 (56.1%)
8. Use Internet games to escape or relieve a negative mood.	793 (35.4%)	1446 (64.6%)	246 (83.7%)
9. Have jeopardized or lost a significant relationship, job, or educational or career opportunity because of participation in Internet games.	197 (8.8%)	2042 (91.2%)	179 (60.9%)

### Examining the associated factors of IGD

In the binary logistic regression, general expenditure per month (501–1000: OR = 0.420; 95% CI = 0.232,0.758; 1001–2000: OR = 0.394; 95% CI = 0.221,0.702), depressive symptoms (10–19: OR = 2.641; 95% CI = 1.985,3.512; 20–30: OR = 4.692; 95% CI = 2.689,8.186) and family and peer support (OR = 0.425; 95% CI = 0.244,0.740) revealed significance in relation to IGD. Adjusted for the significant background variable (i.e., general expenditure per month), depressive symptoms and family and peer support remained significance. Participates who had moderate risk (AOR = 2.670; 95% CI = 2.010,3.547) and high risk depressive symptoms (AOR = 4.770; 95% CI = 2.747,8.283) were more likely to have IGD. In addition, participates who had high level support (AOR = 0.424; 95% CI = 0.245,0.734) were less likely to have IGD than mild level support. It was summarized in Table 3.

**Table 3:** Logistic regression analysis of variables associated with IGD

Variables	OR (95% CI)	AOR (95% CI)
**Grade**		
Freshman	Ref	
Sophomore	1.192 (0.829–1.714)	
Junior	1.166 (0.803–1.694)	
Senior	0.997 (0.651–1.528)	
**Native residential area**		
Big city	Ref	
Small and medium-sized cities	0.834 (0.477–1.459)	
Town	1.343 (0.775–2.329)	
Countryside	1.293 (0.758–2.206)	
**General expenditure per month (CNY)**		
≤ 500	Ref	Ref
501–1000	0.420 (0.232–0.758) **	0.444 (0.247–0.798) **
1001–2000	0.394 (0.221–0.702) **	0.393 (0.222–0.696) **
≥ 2001	0.840 (0.412–1.714)	0.767 (0.380–1.547)
**Depressive symptoms (score ranging 0–30)**		
0–9	Ref	Ref
10–19	2.641 (1.985–3.512) ***	2.670 (2.010–3.547) ***
20–30	4.692 (2.689–8.186) ***	4.770 (2.747–8.283) ***
**Family and peer support (score ranging 0–60)**		
0–20	Ref	Ref
21–40	0.588 (0.337–1.029)	0.596 (0.343–1.035)
41–60	0.425 (0.244–0.740) **	0.424 (0.245–0.734) **

* *p* < 0.05, ** *p* < 0.01, *** *p* < 0.001. AOR: Adjusted odds ratio for the significant background variable (i.e., general expenditure per month) included in this study

### Mediation analysis of family and peer support on the depressive symptoms in relation to IGD

**Fig. 1:**
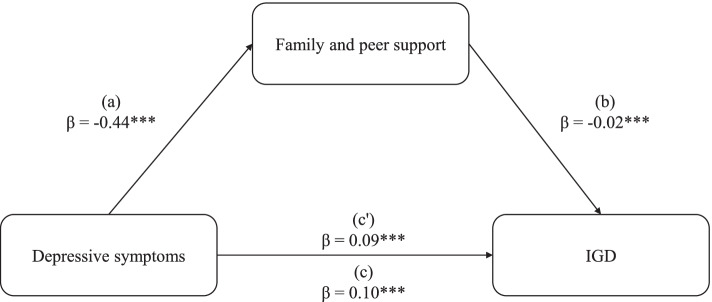
Mediation analysis of family and peer support on the depressive symptoms in relation to IGD

Note. *** *p* < 0.001.

The result of the mediation analysis was to determine whether family and peer support act as a mediating variable between depressive symptom (independent variable) and IGD (dependent variable). As shown in Fig. 1, in the equation (a), depressive symptoms were negatively linked to family and peer support (β = − 0.44, *p* < 0.001). In the equation (b), the mediating variable (family and peer support) was negatively associated with IGD (β = − 0.02, *p* < 0.001). In the equation (c), depressive symptoms have a significant predictive effect on IGD (β = 0.10, *p* < 0.001), and the direct predictive effect is still significant (β = 0. 09, *p* < 0.001, equation c’) when added the mediating variable.

## Discussion

In the present study, we found that the presence of symptoms of IGD among male college students in Nanchong City was 11.6%. Depressive symptoms were identified as a significant risk factor while family and peer support were identified as a significant protective factor associated with IGD. Besides, family and peer support would attenuate the relationship between depressive symptoms and IGD. Several recommendations were provided based on the study findings.

Findings from the present study indicated that the IGD among male college students was higher than most previous reports found among primary school students to high school students (e.g., 3.1% among students from grade four to senior high in Taiwan [29], 5.4% among secondary school students in Thailand [30], 2.5% among eighth graders in Slovenia [31], 1.16% among ninth-grade school adolescents in German [32], 9.2% in high-school students in Lebanese [33]). It was also higher than central regions of China, as Shen Y et al. reported 7.7% among college students in Hunan Province [34]. The probable reason for the results might be that only male students were included in our study. Evidence revealed that IGD is a significant emergent men’s health issue [35]. The majority of research confirmed that males were at higher risk of developing online game addiction [6, 8, 16, 30]. Moreover, college students tended to have fewer academic tasks and less academic pressure, which rendered them more likely to spend too much time online [36]. The regional differences and heterogeneity of diagnostic instruments might also be the explanation of difference between studies. It is worth mentioning that the presence of symptoms of IGD may be somewhat over-estimated, as some researches reveals that the DSM-5 criteria estimates were usually higher than those based on the ICD-11 criteria [6].

In the high presence of symptoms of IGD, the most commonly endorsed items were escapism, continuation and preoccupation both among total participates and the IGD group, which was consistent with previous studies [29]. Indeed, escapism was an essential motivation to use online gaming [37]. Escapism and preoccupation overlapped among many studies as commonly met criteria among all users [29, 31, 38]. This criterion could relate to fantasizing about games and playing games to escape from real-life problems or relieve negative emotional states [27]. Notably, Jesús Castro-Calvo et al. argues that the escapism was judged not relevant in terms of clinical utility, that is to say, it was regarded as incapable of distinguishing between problematic and non-problematic gaming [39]. Therefore, additional research is needed for further study. Moreover, students continue to play even though they aware of the negative consequences of this behavior, which were more likely to be psychosocial than physical in nature [27]. It revealed that enhance the awareness of negative consequences of this behavior might not be the effective prevention way.

Identifying the characteristics of students susceptible to IGD is critical to design targeted intervention programs. In the present study, lower expenditure per month was found to be positively associated with IGD. It was similar to the study of Xue Yang et al. [8], which reported that lower socioeconomic status was significant risk factors of IGD. It was plausible that these students might have higher living stress and self-abasement, which made them indulge in games to relieve stress. Previous research indicated that residential background could discriminate between the adolescents with and without Internet addiction [40]. However, no statistically significant difference in native residential area was found in this study. It could be explained that no matter they came from countryside or big city, they faced the same internet environment in the college.

Additionally, it is confirmed that depressive symptoms were significantly associated with IGD. Evidence revealed that personal mental health status (i.e., depression) was one of the significant predictors for Internet addiction [36]. The individual might feel depressed in the real world and choose to use the virtual world to alleviating their negative mood [30], while excessive use would further intensify depressive symptoms [7]. As the previous evidence showed that Internet addiction and depressive symptoms were potential causes and consequences of each other [41]. Furthermore, individuals exposed to prolonged Internet gaming may be unable to respond to pleasant stimuli as appropriately as healthy individuals due to its impact on individual’s neurocircuitry, possibly yielding the high prevalence of comorbid depression in IGD [42]. Considering the connection between depressive symptoms and IGD, it seemed that depressive symptoms might get masked by IGD sometimes.

Examination of protective factors is also warranted due to many individuals who are at risk for IGD does not develop IGD at end [2]. Our findings revealed that family and peer support was a protective factor to IGD, which was in line with previous studies [8]. During the period of study, families and peers were the important source of support for them to cope with difficulties. Students with higher level of support might have a positive personal development and reduced their boredom and loneliness, which lowered their chance of problematic Internet use when they encountered adversities [43]. Conversely, diminished social support was potential reflections of unfulfilled psychological needs, while inability to fulfill basic psychological needs predicted IGD [5, 44].

In the mediation analysis, this study found that depressive symptoms have significant predictive effect on IGD. After adding the family and peer support variable, the predictive power of depressive symptoms on IGD appeared to decrease. Family and peer support also had a significant predictive effect on IGD. It can be seen that the reduction of the role of depressive symptoms in the prediction of IGD is caused by family and peer support. Therefore, family and peer support can play a role in mitigating the detrimental effect of depressive symptoms on IGD. It has been previously observed that the mediating effects of social support exists in other psychological states [5, 45]. The study highlights that reinforcing family and peer support for college students with depressive symptoms may reduce to develop IGD, that is, family and peer support have the buffering effects. Nevertheless, the buffering effects of family and peer support should not be overestimated, because the effect size was quite small, though the mediating effects of family and peer support on the relationship between depressive symptoms and IGD was statistically significant.

The high presence of symptoms of IGD needs warrant interventions, which has lasting harmful effects on college students. Chinese authorities need to allocate more resources of IGD prevention to less developed areas, such as increasing funding for IGD-related prevention programs. Public health policies should increase their focus on the IGD of the public. On the college level, it is clamant to strengthen school psychology guidance to release students’ pressure. Also, schools may systematically train teachers and act as informational and screening hubs for early signs or stages of problem behaviors [46]. Additionally, evaluation for psychosocial status (i.e., depressive symptoms) may be added in assessment of students suspected of IGD to enhance the screening and treatment capabilities. Furthermore, improving parent-child communication and promoting a good quality of family and peer relationship may be a promising approach in preventing and reducing IGD.

There are several limitations that should be noted. Firstly, the current sample was collected in one city, limited generalization to other places. Secondly, in order to reduce the questionnaire length and response time, we only collected a limited number of variables. Some potential influencing factors (e.g., anxiety or substance use) were not included and they may assist the development of effective interventions. And we used simplified scales to identify IGD, even demonstrated adequate reliability and validity, a more comprehensive inquiry would enrich the outcomes. Electronic measurement of Internet gaming pattern may offer a more objective measure to investigate Internet gaming behaviors (e.g., frequency of gaming, duration of gaming). Future studies can include such measures to better understand Internet gaming behaviors among college students. Thirdly, the information about family and peer support was obtained using self-administered questionnaires, in which may conceal a possibility of subjective bias. At last, the design to this study was cross-sectional, limiting the ability to address causality or direction of the associations observed. Additionally, all data were self-reported, and susceptible to self-report bias, though we assured the respondents of anonymity and encouraging them to be frank in making their response. The study also has several strengths. With 2533 male college students, this study has the relatively large sample size and exhibit the current situation of IGD in underdeveloped western area in China.

## Conclusion

In summary, this study found that one in ten male college students reported clinically significant IGD symptoms, which indicate that IGD is an important public health problem in Nanchong, China. Family and peer support would attenuate the relationship between depressive symptoms and IGD. The results highlighted the importance that interventions for IGD should not only pay attention to personal mental health status (e.g., depressive symptoms), but also promote support. A more integrated and holistic intervention in a combination of psychological and social aspects is needed to address IGD among college students.

## Data Availability

The original data are available upon request to the corresponding author.

## Abbreviations

IGD: Internet gaming disorder
DSM-5: The fifth edition of the Diagnostic and Statistical Manual for Mental Disorders
GFI: Goodness of fit index
AGFI: The adjusted goodness of fit index; NFI: Normed fit index
TLI: Tucker Lewis Index
RMSEA: Root mean square error of approximation
CESD-10: The Short form of the Epidemiologic Studies Depression Scale
SD: Standard deviation
CNY: China Yuan
AOR: Adjusted odds ratio
ICD-11: The 11th revision of the International Classification of Diseases
